# Forensic Toxicological Aspects of Misoprostol Use in Pharmacological Abortions

**DOI:** 10.3390/molecules27196534

**Published:** 2022-10-03

**Authors:** Paweł Szpot, Olga Wachełko, Marcin Zawadzki

**Affiliations:** 1 Department of Forensic Medicine, Wroclaw Medical University, 4 J. Mikulicza-Radeckiego Street, 50345 Wroclaw, Poland; 2 Institute of Toxicology Research, 45 Kasztanowa Street, 55093 Borowa, Poland

**Keywords:** misoprostol, misoprostol acid, pharmacological abortion, miscarriage, fetus, UHPLC-QqQ-MS/MS, forensic toxicological investigations

## Abstract

The aim of this study was establishment of an UHPLC-QqQ-MS/MS method for the deter-mination of misoprostol acid in biological specimens in cases of pharmacological abortions. Forensic toxicological examination was performed in three different biological samples (whole blood, placenta and fetal liver). The validation parameters of the method were as follows: limit of detection: 25 pg/mL; limit of quantification: 50 pg/mL, coefficient of determination: >0.999 (*R^2^*), intra- and interday accuracy and precision: not greater than 13.7%. The recovery and matrix effect were in the range of 88.3–95.1% and from −11.7 to −4.9%, respectively. Toxicological analysis of the mother’s blood (collected two days after pregnancy termination) did not reveal any abortifacients; however, misoprostol acid was found in the placenta (793 pg/g) and fetal liver (309 pg/g). The second case involved a fetus found near a garbage container. The concentration of misoprostol acid in the placenta was 2332 pg/g. In the presented study, an extensive literature review of misoprostol pharmacokinetics studies was performed. To our knowledge, the UHPLC-QqQ-MS/MS technique presented in this paper is the first quantitative method applied for forensic toxicological purposes. In addition, postmortem concentrations of misoprostol acid in miscarried fetuses due to illegal abortions were reported for the first time.

## 1. Introduction

**Figure 1:**
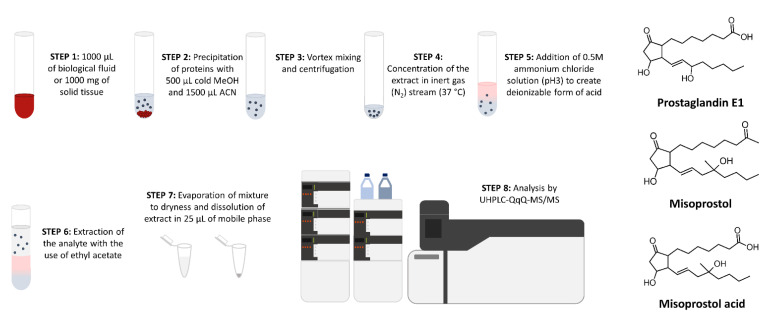
Scheme of biological samples preparation and analysis by developed UHPLC-QqQ-MS/MS as well as chemical structures of prostaglandin E1, misoprostol (synthetic PGE1 analogue) and its main metabolite–misoprostol acid.

The World Health Organization (WHO) defines unsafe abortion as a “procedure for terminating an unintended pregnancy carried out either by a person lacking the necessary skills or in an environment that does not conform to minimal medical standards, or both” [1]. During 2010–2014, 55.7 million abortions were performed each year. 30.6 million were safe, 17.1 million were less safe, and 8.0 million were the least safe. This means that 25.1 million abortions each year were not performed with proper safety measures. The percentage of unsafe abortions was much higher in developing countries than in developed countries (49.5% vs. 12.5%) [2]. Data show that in 2015–2019 there were 121.0 million unintentional pregnancies per year. A total of 61% of these pregnancies ended in an abortion, which gives a large number of over 70 million abortions each year [3]. An estimated 6.9 million women were treated for complications from unsafe termination of pregnancy in 2012 [4]. Deaths due to an unsafe abortion remain close to 13% of all maternal deaths [1]. Considering the above-mentioned data, the worldwide problem of unsafe abortion seems to be really important. One method of pregnancy termination is the use of abortion pills in hospitals. These drugs are still not available in many countries, so women who want to carry out pharmacological abortion with abortion pills usually look for help on websites and online marketplaces. Such medications consist of mifepristone and misoprostol [5]. Mifepristone (RU-486) is known as a competitive inhibitor of progesterone, and it was first synthesized and widely distributed in the 1980s [6]. Misoprostol is a prostaglandin E1 (PGE1) analogue developed for gastric ulcer prevention but is commonly used in reproductive health (Figure 1). Misoprostol causes cervical softening and dilation, as well as uterine contractions. Indications for use of misoprostol include: management of postpartum hemorrhage, incomplete abortion, first and second trimester medical abortion, induction of labour and cervical ripening. Routes of administration of this drug include oral, vaginal, rectal, buccal and sublingual [7]. Trade names of preparations containing misoprostol are e.g., Arthrotec, Arthrotec forte, Cytotec, Misotrol, Gymiso, Prostokos, Vagiprost and Oxaprost. After administration, misoprostol is rapidly absorbed and converted into its pharmacologically active metabolite, misoprostol acid. After oral ingestion, plasma concentrations of misoprostol acid peak in approximately 30 min and decline rapidly thereafter. If the misoprostol is administered vaginally, the peak plasma concentration of misoprostol acid is reached in 1–2 h and then declines slowly [8]. The therapeutic dose of misoprostol is very small, usually 400–800 µg daily, meaning that the highest concentration of the active metabolite is very low (below 1 ng/mL), and therefore, a sensitive method for the quantification of misoprostol acid is necessary. An example of a hazardous method of terminating a pregnancy may be the use of abortion pills of an unknown origin (bought on the Internet) by a pregnant woman and inducing the abortion by herself at home without medical supervision or specialist care. The consequences of such a practice can be dangerous. Misoprostol may increase the risk of uterine rupture [9,10,11], massive intraperitoneal hemorrhage, hemodynamic instability and hysterectomy [12]. Fatal toxic shock secondary to infection after medical abortion with mifepristone and misoprostol may develop [13,14]. Moreover, overdoses of misoprostol during pregnancy have also been reported, one of which ended in death [15]. Manifestations of toxicity included hypertonic uterine contraction with fetal death, hyperthermia, rhabdomyolysis, hypoxemia, respiratory alkalosis and metabolic acidosis [16]. In a second case, manifestations included abdominal pain, vomiting, diarrhea and confusion [17]. In a third case, manifestations included fever, tremor, tachycardia, hypertension, nausea and abdominal cramping [18]. As far back as the 1990s, it was pointed out that the access to medical information from unregulated sources through the Internet would increase the potentially dangerous use of misoprostol as an abortifacient [19]. Even today there is not enough data to be able to determine the safety of abortion pills from the Internet, so more research is needed. As the number of illegal abortions increases, counterfeit abortifacients are becoming more widely available on the black market. Considering that the concentration of active ingredients can vary dramatically between different fake abortion-inducing drug specimens, their illegal distribution can pose a significant public health problem [20]. Misoprostol (one of the active ingredients of abortion pills) is used to induce an abortion during an unwanted pregnancy, which is a crime according to the law in some countries. This aspect is particularly important in forensic toxicology, since in some countries misoprostol is not approved [7]. In such cases it would be necessary to test the blood of women who have had miscarriages (or blood of dead infants) to confirm or exclude the possibility of using this drug. In the literature we were not able to find any information regarding concentrations of misoprostol acid in the fetal biological material or in placentas tested in forensic toxicology laboratories in cases of pregnancy termination. The data in the available literature concerning methods for the determination of misoprostol acid in biological material describe using a gas chromatography tandem mass spectrometry (GC-MS/MS) [21] and high-performance liquid chromatography tandem mass spectrometry (HPLC-MS/MS) [22,23,24,25,26]. Watzer et al. detected misoprostol acid in vomit and urine by GC-MS/MS and LC-MS/MS, but only qualitatively [27]. The aim of this study was to develop and validate a method for the determination of misoprostol acid in whole blood, placenta and fetal liver using ultra-high-performance liquid chromatography coupled with triple quadrupole mass spectrometry (UHPLC-QqQ-MS/MS). Furthermore, the available results of clinical studies on the concentrations of misoprostol acid in various biological materials in the literature were summarized. Such data collection resulted in the development and optimization of the UHPLC-QqQ-MS/MS method as well as sample preparation procedure for forensic toxicology investigations. Moreover, two cases of abortifacient ingestion for self-induced pregnancy termination were described. The method presented in this paper was applied for the first time for determination of misoprostol acid in postmortem biological tissues and fluids.

### Case History

- Case 1

A woman (age and medical history unknown), having two children, as soon as she realized she was pregnant, began to search for information on the Internet about how to terminate a pregnancy with abortion pills. The woman found a forum that described the effects of using Arthrotec^®^. The woman bought the pills online at an auction website. The seller told her that the pills were safe and would end a pregnancy lasting up to twelve weeks (recommended dosage was 4 tablets every 4 h). On the day before the miscarriage, at around 9 p.m., the woman took four Arthrotec Forte^®^ tablets vaginally. She started feeling unwell and sick and had abdominal pain. After four hours (1 a.m.), she took another four tablets of the drug orally. After the fourth dose she began to have uterine contractions but still no bleeding or spotting. After the fifth dose, a miscarriage occurred. The mother’s blood was collected for toxicological examination two days after taking the last dose of pills. An external and internal examination of the fetus was performed three days after the miscarriage. During the autopsy, samples were collected for histopathological examination, as well as placenta and liver for toxicological investigations.

- Case 2

On the sidewalk next to the garbage container a male human fetus was revealed (3-4 months of pregnancy), covered with clothes. There was no cardiac or respiratory activity, but there were several recognizable signs of death, such as rigor mortis. Three days after the corpse was found, an autopsy was performed during which the placenta was secured for toxicological examinations.

## 2. Materials and Methods

### 2.1. Chemicals and Reagents

Water (Chromasolv^®^ LC–MS), acetonitrile (Chromasolv^®^ LC–MS), methanol (Chromasolv^®^ LC–MS), ethyl acetate and formic acid were purchased from Sigma-Aldrich (Steinheim, Germany); ammonium formate was purchased from Sigma-Aldrich (Mumbai, India); ammonium chloride was purchased from Merck (Saint Louis, MO, USA) and misoprostol acid and misoprostol acid-*d_5_* were purchased from Toronto Research Chemicals INC (Toronto, ON, Canada). Standard solutions of misoprostol acid and misoprostol acid-*d_5_* were prepared in methanol. The standard solutions were stored in a refrigerator at −20 °C.

### 2.2. Instrumentation

Chromatographic analysis was performed using an ultra-high-performance liquid chromatograph (UHPLC Shimadzu Nexera LC-40 System, Kyoto, Japan). The separation was done using an ACQUITY UPLC BEH C18 1.7 µm 2.1 × 100 mm (Waters Corp., Milford, MA, USA) column with a thermostat at 40 °C. A mixture of 10 mM ammonium formate/0.1% formic acid in water (A) and 10 mM ammonium formate/0.1% formic acid in methanol (B) was used as a mobile phase. The gradient elution was carried out at constant flow of 0.3 mL/min. The gradient applied was the following: 0 min–5% B, 7.5 min–95% B and then 10 min–95% B. A return to started gradient compositions (95% A and 5% B) was performed for 5 min. The injection volume was 10 μL. Detection of the investigated compounds was achieved using a triple quadrupole mass spectrometer (QqQ, Shimadzu 8060, Kyoto, Japan). The spectrometer was equipped with an electrospray (ESI) source; determination of the investigated substances was carried out in the multiple reaction monitoring (MRM) mode (negative ionization). The following MS parameters were fixed: nebulizing gas flow: 3 L/min; heating gas flow: 10 L/min; interface temperature: 300 °C; desolvation temperature: 526 °C; DL temperature: 250 °C; heat block temperature: 400 °C and drying gas flow: 10 L/min. A summary of precursor and product ions, collision energies, dwell time, Q1–Q3 pre-bias voltages and retention time for each compound is presented in Table 1.

### 2.3. Blank Material

Blank samples of human postmortem blood were collected during autopsies performed in Department of Forensic Medicine. Blank samples were screened prior to spiking to ensure that they were free from misoprostol acid. Authentic biological samples collected in two forensic cases were sent to our laboratory for toxicological analyses. Biological fluids were collected in tubes with sodium fluoride, and solid tissues were collected in plastic containers (without any preservative agent).

### 2.4. Sample Preparation

Human postmortem blood (1000 μL) or tissue homogenate (1 g) was transferred to a 12-mL plastic tube, adding 10 μL internal standard (misoprostol acid-*d_5_* at a concentration of 100 ng/mL). The samples were vortex-mixed, followed by drop-wise addition of 500 μL of cold methanol and 1500 μL acetonitrile, and then mixed for 30 s. Then samples were centrifuged at 1520× *g* for 10 min. Supernatant was transferred into a 12-mL plastic tube and evaporated to the volume of 500 μL in a stream of nitrogen at 37 °C. Next, the extract was mixed with 500 μL of 0.5 M ammonium chloride solution (pH3). Liquid–liquid extraction with ethyl acetate (2 mL) was carried out for 10 min. Samples were centrifuged at 1520× *g* for 10 min and the organic phases (about 2 mL) were transferred to 2-mL Eppendorf tubes and evaporated to dryness under a stream of nitrogen (at 37 °C). The extracts were dissolved in 25 μL of mobile phase, transferred to the glass insert and analyzed by UHPLC-QqQ-MS/MS. The summary of all preanalytical steps is presented in Figure 1.

### 2.5. Working Solutions, Calibration Curve, Quality Control Samples

Standard solutions were diluted with methanol to obtain working standard solutions at the following concentrations of misoprostol acid: 0.5, 1, 2, 5, 10, 20, 50, 100 and 200 ng/mL. Calibration points and quality control samples (QC) were prepared by diluting the appropriate working solution with human blood. The final concentrations of the calibrators were: 50, 100, 200, 500, 1000, 2000, 5000, 10 000 and 20 000 pg/mL blood for misoprostol acid. QC samples were prepared by spiking blank human blood to yield final concentrations of 100 (low QC), 1000 (medium QC) and 10 000 (high QC) pg/mL for misoprostol acid.

### 2.6. Validation

Validation of the method included examination of selectivity; linearity; precision and accuracy; carryover; limit of detection (LOD) and quantification (LOQ); recovery and matrix effect. Selectivity of the method was established by analyzing five different lots of blank blood for possible endogenous interference peaks at the retention time of the misoprostol acid. Linearity was evaluated by an analysis of misoprostol acid working solutions with blank whole blood in the calibration range of 50–20,000 pg/mL. A linear calibration model was applied. The coefficient of determination (*R^2^*) was determined. The precision and accuracy were estimated by replicating analysis (*n* = 5) of QC samples at three concentration levels: 100 (low QC), 1000 (medium QC) and 10 000 (high QC) pg/mL. Precision was defined as relative standard deviation (RSD%), whereas accuracy was expressed as mean relative error (RE%). Intraday precision and accuracy were evaluated by analyzing QC samples five times over 1 day, whereas interday values were estimated by analyzing QC samples five times on five different days. To investigate the carryover, three samples without analytes were analyzed after a calibration sample at the upper limit of quantification (ULOQ). Unacceptable carryover was when a peak area ratio in a zero sample after analysis of a sample containing a high concentration of misoprostol acid exceeded 20% of the area ratio observed for the LOQ samples. The LOQ was defined as the concentration at which the relative standard deviation (RSD%) does not exceed 20%, and the signal-to-noise ratio met the minimum condition: S/N ≥ 10. The LOD was considered to be the lowest concentration of the sample for which the signal-to-noise ratio met the minimum condition: S/N ≥ 3. The recovery (*n* = 5) was evaluated at each of three concentration levels: 100 (low QC), 1000 (medium QC) and 10 000 (high QC) pg/mL. The recovery (in percent) was determined by comparing the response of extracted analytes in a spiked blank biological specimen versus the response of standard solutions. The matrix effect (in percent) was calculated using an equation described by Chambers et al. [28].

## 3. Results

### 3.1. Method Development

**Figure 2:**
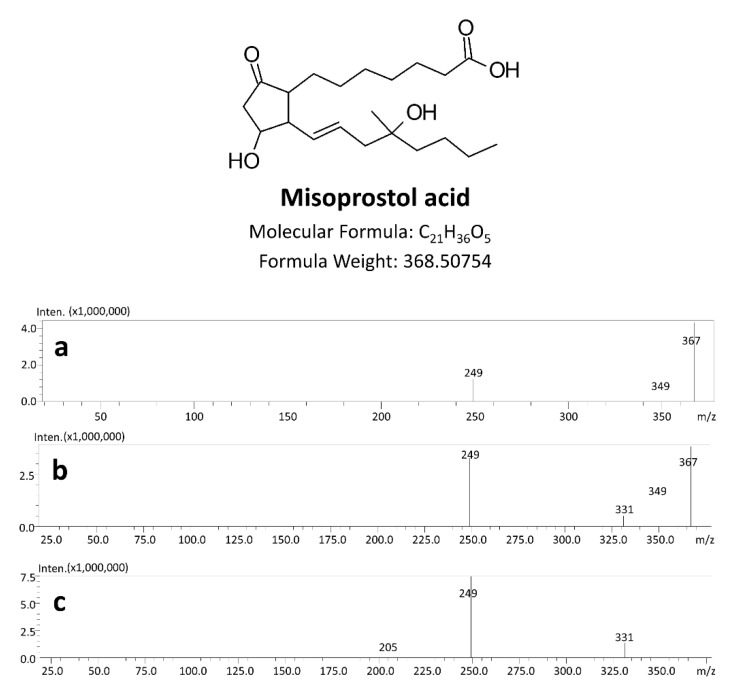
Product ion scan spectra of misoprostol acid at different collision energies: (**a**) 5 V; (**b**) 10 V and (**c**) 20 V.

In order to select the most suitable solvent for misoprostol acid extraction, chloroform, dichloromethane, diethyl ether, hexane and ethyl acetate were examined (unpublished data). The highest peak area was observed in case of ethyl acetate, and therefore this organic solvent was chosen for the method establishment. The presented method was developed using tandem mass spectrometry in MRM mode in negative ionization mode. The product ion scan spectra of misoprostol acid are presented in Figure 2. The *m/z* transitions of 367.0→249.1 (misoprostol acid) and 372.5→249.0 (misoprostol acid-*d_5_*) were selected as the most optimal for the quantitative analysis. In the presented UHPLC-QqQ-MS/MS method, additional MRM transitions were monitored: 367.0→331.05 *m/z* and 367.0→349.1 *m/z* for misoprostol acid as well as 372.5→336.05 *m/z* and 372.5→354.1 *m/z* for misoprostol acid-*d_5_*. However, it is worth noticing the fact that in the above-mentioned case of misoprostol acid, the transitions in the complex matrix (e.g., postmortem whole blood) have low signal-to-noise ratios and are not suitable as confirmation transitions in the determination of trace concentrations of misoprostol acid. The threshold concentration for these MRM transitions is 5 ng/mL, and above this level the S/N ratio is found to be acceptable.

### 3.2. Validation Results

**Figure 3:**
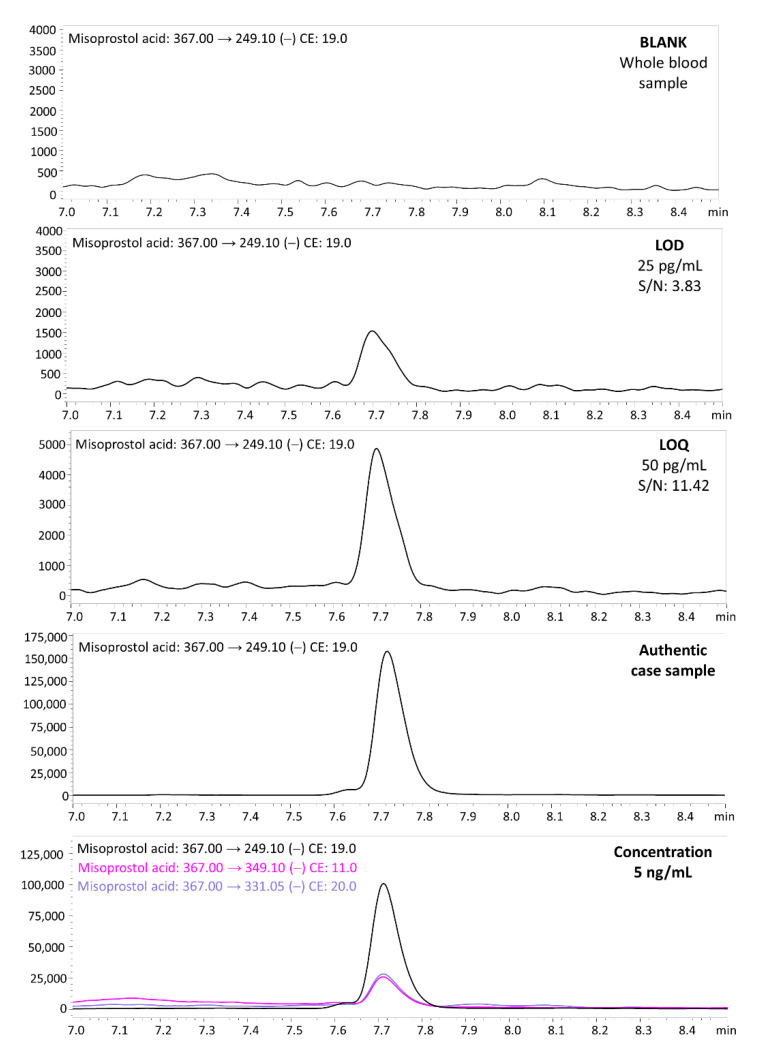
Chromatograms of the blank whole blood sample; misoprostol acid in blood at a concentration of LOD (25 pg/mL); misoprostol acid in blood at a concentration of LOQ (50 pg/mL); chromatogram of authentic placenta sample (Case 2); and misoprostol acid in whole blood at the concentration of 5 ng/mL (with all monitored MRM transitions).

In the described method, very good validation parameters were achieved. The LOD was 25 pg/mL (S/N: 3.83; RE 13.3%) and the LOQ was 50 pg/mL (S/N: 11.42; RE 10.7%). The coefficient of determination was >0.999 (*R^2^*). The intra- and interday recovery and matrix effect values are summarized in Table 2. Presented values are in accepted ranges in accordance with GTFCh (Gesellschaft für Toxikologische und Forensische Chemie ang. German Society of Toxicological and Forensic Chemistry) recommendations. There were no substances carried over between samples. All peaks were well separated, and no endogenous substances interfered with the retention times of the analyte or internal standard. Precision and accuracy values did not exceed 13.7%. Recovery and matrix effect values were in the range of 88.3–95.1% and from −11.7 to −4.9%, respectively. Chromatograms of the blank sample, misoprostol acid in whole blood at a concentration of LOD and LOQ, chromatogram of an authentic sample (Case 2) as well as misoprostol acid in whole blood at the concentration of 5 ng/mL (with all monitored MRM transitions) are presented in Figure 3.

### 3.3. Autopsy, Histopatological and Toxicological Findings

- Case 1

The autopsy revealed a female fetus with no malformations or lesions and an unaltered afterbirth (i.e., placenta, umbilical cord and fetal membranes). Among pathological findings, only hemorrhagic petechiae were found in the subcutaneous tissue of the head. Based on the results of the postmortem examination, it should be concluded that the fetus could have been born alive (positive lung flotation test); however, due to extreme prematurity, the fetus was not capable of autonomous postnatal life. The pregnancy termination occurred at 21 weeks. Histopathological examination revealed: congestion and cerebral edema; signs of immaturity of the cerebral cortex; single petechial hemorrhages; erythropoietic foci of the spleen; congenital pulmonary airway malformation, i.e., congenital cystic adenomatoid malformation type 3 (CCAM); multiple erythropoiesis foci in the liver and placental hemorrhagic hyperplasia. Toxicological analysis of the mother’s blood (collected two days after pregnancy termination) did not reveal any drugs, psychotropic substances or abortifacients. The placenta contained the following substances: diclofenac (27.6 µg/g), misoprostol acid (793 pg/g) and ethyl alcohol (0.44 mg/g). The fetal liver contained: diclofenac (2.2 µg/g), misoprostol acid (309 pg/g) and ethyl alcohol (0.30 mg/g).

- Case 2

The corpse of a male fetus was connected by an umbilical cord to the afterbirth, physique appropriate to fetal age, poor nutrition, weight 34 g, body length 12.3 cm, corresponding to 3–4 months of pregnancy. No malformations or lesions were revealed. However, characteristics of an intrauterine fetal maceration were found: desquamation with formation of bullae and skin peeling, and laxity of the cranial sutures and ligaments. Histopathological examination revealed: visible signs of atelectasis and, in the placenta, widespread prenatal hemorrhages dominating within the maternal section. The majority of organs presented signs of partial autolysis. Toxicological analysis of the placenta revealed diclofenac at a concentration of 305.6 µg/g and misoprostol acid in a concentration of 2332 pg/g. Diclofenac concentrations in the above-mentioned biological materials collected in both cases were quantified by the method described earlier by Szpot et al. [29].

## 4. Discussion

To date, techniques such as: HPLC-MS [22,23,25,30] and UHPLC-MS [24,26] have been developed. The detectors that make it possible to develop highly sensitive and selective methods are triple quadrupole mass spectrometers (QqQ-MS/MS) operating in MRM mode. In all the cited methods, negative ionization was applied, and in the majority of methods, only one MRM transition was monitored: 367 *m/z*→249 *m/z*. Unfortunately, monitoring a single MRM transition in a complex matrix such as, for example, whole blood samples or placenta makes it necessary to carry out an efficient extraction during the preanalytical step and to purify the extract as well as possible. The investigations performed to date revealed that both liquid–liquid extraction (LLE) using diethyl ether and dichloromethane [22] or ethyl acetate and toluene [25] as well as solid-phase extraction (SPE) with the use of Oasis^®^ HLB [24,30] and Oasis^®^ MAX [23,26] can be successfully used for this purpose. Furthermore, the use of a deuterated analog of misoprostol acid makes it possible to achieve recovery values of more than 80% [23,25,30]. Determination of misoprostol acid and its metabolites is also possible using GC-CI-QqQ-MS/MS in negative ionization mode [21]; however, the sample preparation in the above-mentioned method is very complicated and time-consuming (involving two different derivatizations techniques). Considering the unavailability of information in scientific papers regarding the concentrations of misoprostol acid in postmortem samples as well as guidelines for the analytical methods used to confirm maternal misoprostol intake, the authors decided to collect clinical data to facilitate the development of an appropriate procedure in our laboratory. From the toxicological forensic perspective, several different issues are important, as shown in the table (Table 3). After analyzing the presented data, it is noteworthy that the average concentrations of misoprostol acid after a single intake in the biological material were in the range of 27.2─2683.0 pg/mL, and the mean time of maximum concentration (Tmax) was in a range of 14.2─120 min. It should be noted that the concentrations of misoprostol acid in postmortem material found in this study are also within the range of the aforementioned values. In clinical studies, the determination of misoprostol acid was performed either in plasma or serum. After taking into consideration all the details presented in Table 3 (except for slow-release tablets and tablets taken together with saline solution or vinegar solution), it should be noted that higher concentrations of misoprostol acid were observed in serum than in plasma, in all cases. Consequently, it can be concluded that serum is probably a far better biological material for toxicological examination than plasma. Misoprostol acid is also detectable in breast milk. Its maximum concentration is 7.6 ± 2.8 pg/mL 1.1 h after taking a single 200 µg dose of misoprostol. After 5 h, the concentration in breast milk drops to a concentration of 0.2 pg/mL [31]. Such data would be crucial if a breast milk sample were sent to a toxicology laboratory for investigation regarding the presence of abortifacients.

Misoprostol acid is unstable in whole blood at room temperature. Its concentration decreases by 90% within 16 days and after a month it becomes undetectable. Freezing the sample reduces the degradation rate of misoprostol acid [24]. In addition, degradation of misoprostol acid can also occur during the pre-analytical process. Misoprostol acid concentration in the already prepared sample can degrade by 20% at room temperature within 12 h [22]; therefore, it is important to carry out analysis as quickly as possible after sample preparation. Other studies suggest that misoprostol acid in plasma is stable at room temperature for 23 h [30]. The problem of the instability of misoprostol acid in biological material is especially important when testing postmortem material. The corpses of fetuses in cases of illegal abortion could be found, for example, on the sidewalk (Case 2), in garbage cans, sewage farm [29], etc., where external conditions are highly variable and tanatochemical processes can lead to the degradation of a misoprostol acid. Cases of misoprostol use for abortion purposes by women without specialized medical care (as described in this paper) confirm that, besides the enormous risks, such a hazardous method of pregnancy termination is unfortunately still common (especially in countries with restrictive abortion laws). Once a pregnancy is terminated, it is generally very difficult to potentially confirm a woman’s use of abortifacients. The abortion performed can often be revealed from the testimony of the patient who terminated the pregnancy, sometimes from witnesses with whom she corresponded about it or from medical personnel to whom the patient came with heavy bleeding or with miscarried fetus. Sometimes a gynecological examination can reveal the presence of pills in a woman’s reproductive tract. Such a case was described by Hopson and Ross [41]. In this case, three pills were found in the vagina, but these medications were not collected for toxicological analysis. The results of histopathological examinations of the material collected during the postmortem examination of the female fetus (Case 1) revealed a congenital airway malformation of the lungs, i.e., congenital cystic adenomatoid malformation type 3 (CCAM). CCAM is characterized by multicystic lung tissue resulting from a proliferation of bronchial structures that impairs alveolar development and function. The lung parenchyma is replaced by nonrespiratory tissue leading to the formation of cysts of various sizes [42]. Pathologists in all the cases described in this paper concluded that due to extreme prematurity, the fetuses (Case 1 and Case 2) were incapable of independent extrauterine life. Another noteworthy observation is that in all the cases described in this paper, along with misoprostol/misoprostol acid, another substance found in toxicological studies was diclofenac. On this basis, we can hypothesize that the pills used to induce miscarriage could be Arthrotec^®^ or Arthrotec forte^®^, as these drugs contain both active substances (misoprostol and diclofenac) and are the only such drugs available in our country. It is also worth noticing the fact that the forensic toxicological aspect of abortions is still not a well-investigated knowledge domain. Although Hopson and Ross [41] described as many as five forensic toxicology cases in which early abortion pills were used, they did not present toxicological data supporting this information. Scientific papers provide no information on concentrations of misoprostol acid in the blood of fetuses, placentas or internal organs of fetuses. Furthermore, the collection of pills for further testing (which are found during a gynecological examination) is not a common practice, and even if the material would be secured for forensic examinations, there is the additional problem associated with targeted analysis. Routine toxicology screening focuses mainly on detecting substances with high potential for poisoning, including illicit drugs (e.g., new psychoactive substances) and pharmaceuticals. Abortifacients or emergency contraceptives are not routinely determined substances in toxicology laboratories. In the case of misoprostol acid quantification, factors such as rapid metabolism, extreme instability or the need for implementation of special sample preparation techniques make detection of this substance in the biological samples even more difficult. Another point worth emphasizing is the histopathological examination of the placenta, which should exclude the possibility that the miscarriage occurred due to placental causes. An extensive article has been written about how to secure the placenta for histopathological examination and its significance [43]. All of the aspects discussed above show how difficult it is to compile adequate evidence to prove a direct connection between the use of abortion pills and miscarriage and/or fetus death. Although misoprostol is considered to be a safe drug when pharmacological abortions are performed in the hospital, there are a number of dangers associated with the use of this substance without medical supervision (the hazards related with such a practice are described in detail in the introduction). It is also worth noting that in the literature there is one fatal case of misoprostol usage. A teenage girl was supposed to have taken misoprostol at a dose of 800 µg orally every 2 h, with night breaks, for 2 days (she took a total amount of 60 tablets i.e., 12 mg). A laparotomy was performed. Extensive necrosis of the lesser curvature of the stomach and distal part of the esophagus was found [15]. The authors indicated that the mechanism causing the involvement of misoprostol in gastrointestinal ischemia and necrosis is unknown; however, it is worth mentioning that there was no information in the article about performing extensive toxicological studies to confirm the pills’ composition. Confirming that the drug used by the patient contained only misoprostol would be a key factor in excluding the possibility that any other substance caused the gastric necrosis. In addition, it is still worth noting that drugs such as Arthrotec forte^®^ (Case 1), for example, were developed exclusively for oral use. However, these tablets have been used orally as well as rectally, vaginally and, more recently, sublingually. Nonetheless, there are no data on the safety of misoprostol applied by other routes, so it can be concluded that such studies should be conducted in the future.

## 5. Conclusions

The developed and fully validated ultra-sensitive UHPLC-QqQ-MS/MS method for misoprostol acid determination was successfully applied in two authentic forensic cases (analysis was performed in three different matrices: whole blood, placenta and liver). Toxicological analysis of the mother’s blood did not reveal any abortifacients. Misoprostol acid was found in other biological specimens at a concentration of 793 pg/g in the placenta (Case 1) and 2332 pg/g (Case 2), as well as in fetal liver at a concentration of 309 pg/g. The acquired LOQ value (50 pg/mL) allows very sensitive determination of misoprostol acid in cases of pharmacological abortions, and it can be concluded that the presented method is suitable and very desirable, especially in forensic toxicology practice. In addition, the LOD value (25 pg/mL) allows for trace analysis of biological specimens. The intra- and interday accuracy and precision did not exceed 13.7%. The recovery and matrix effect were in the range of 88.3–95.1% and from −11.7 to −4.9%, respectively. Unfortunately, despite the use of a highly advanced instrumentation, it was not possible to provide sufficient S/N ratios for the following MRM transitions: 367.0→331.05 *m/z* and 367.0→349.1 *m/z* (which had very low intensities). However, with technological progress and the further evolution of more sensitive and selective mass spectrometers, it may become possible to use three MRM transitions for the determination of misoprostol acid, which will definitely improve the quality of toxicological examinations in the future. In addition, the sample volume may also be reduced as a result of the above-mentioned factors, as well as as a result of implementation of more selective extraction methods (e.g., dedicated SPE columns for misoprostol acid analysis). The advantage of the research presented in this article is the possibility of confirming the use of abortifacients by the mother to self-induce termination of a pregnancy. The developed UHPLC-QqQ-MS/MS method is suitable for the examination of the very complex biological matrix, such as fetal tissues and placenta. This fact is particularly important in case of toxicological analysis of fetuses, from which it is not possible to collect sufficient volume of biological fluids. To our knowledge, the technique presented in this paper is the first quantitative method applied for forensic toxicological purposes (analysis of postmortem fetal samples). In addition, postmortem concentrations of misoprostol acid in miscarried fetuses due to illegal abortions were reported for the first time. On the basis of the literature review and the investigations performed in this study, the following conclusions can be drawn:

- Misoprostol acid passes through breastmilk;
- Average concentrations of misoprostol acid in clinical studies (biological specimens collected from women), depending on the route of administration, are in the range of 27.2─2683 pg/mL;
- Misoprostol acid is highly thermally unstable;
- Misoprostol is rapidly converted into misoprostol acid, which is rapidly eliminated from the body;
- Approximately two days after taking misoprostol (at the dose as in Case 1), misoprostol acid is not determinable in maternal blood due to progressive metabolism and/or degradation processes;
- The developed method allows detection of misoprostol acid in blood at concentrations as observed in clinical studies;
- Concentrations of misoprostol acid in postmortem materials were in range of 309─2332 pg/mL.

## Figures and Tables

**Table 1:** UHPLC–ESI-QqQ-MS/MS parameters for misoprostol acid and misoprostol acid*-d_5_*.

Compounds	Retention Time (min)	Precursor Ions (*m/z*)	Product Ions (*m/z*)	Dwell Time (ms)	Q1 Pre Bias (V)	CE (V)	Q3 Pre Bias (V)
Misoprostol acid	7.71	367.0	249.1 *	30	19	19	12
			349.1		26	11	12
			331.05		13	20	12
Misoprostol acid-*d_5_*	7.70	372.5	249.0 *	30	14	17	19
			354.1		26	11	12
			336.05		13	20	12

* Ions selected for quantitative analysis.

**Table 2:** Validation parameters of the UHPLC-QqQ-MS/MS method for determination of misoprostol acid in postmortem whole blood samples.

Validation Parameters						
Concentration Level [pg/mL]	Intraday		Interday		Recovery [%]	Matrix Effect [%]
	Precision [%]	Accuracy [%]	Precision [%]	Accuracy [%]		
100	13.0	5.6	5.9	13.0	88.3	−11.7
1000	12.2	2.0	9.7	13.7	95.0	−5.0
10000	6.7	2.8	6.7	2.4	95.1	−4.9

*n* = 5.

**Table 3:** Pharmacokinetic parameters of misoprostol acid in clinical trials.

Dose (Route)	Number of Participants	Type of Biological Sample	Mean Time of Maximum Concentration (Tmax) [Time Min]	Mean Maximum Drug Concentration (Cmax) [Pg/Ml]	Ref.
400 µg (oral)	45	human plasma	15.0 ± 4.8	777.74 ± 259.80	[30]
200 µg (oral)	10	human milk	66.0 ± 12.0	7.6 ± 2.8	[31]
400 µg (sublingual)	10	human plasma	26.0 ± 11.5	574.8 ± 250.7	[32]
400 µg (oral)	10		27.5 ± 14.8	287.6 ± 144.3	
400 µg (vaginal)	10		72.0 ± 34.5	125.2 ± 53.8	
400 µg (vaginal+water)	10		75.0 ± 31.6	162.8 ± 57.1	
400 µg slow release (oral)	10	human plasma	54.0 ± 46.5	27.2 ± 14.5	[33]
800 µg slow release (oral)	11		81.8 ± 111.5	43.5 ± 17.7	
400 µg (oral)	10		36.0 ± 12.6	186.2 ± 118.8	
400 µg (oral)	9	human plasma	14.2 ± 7.0	258.7 ± 83.8	[34]
400 µg (rectal)	9		71.7 ± 23.5	86.8 ± 44.7	
400 µg (vaginal)	9		65.0 ± 21.2	210.8 ± 63.0	
400 µg (oral)	10	human plasma	34.0 ± 17.0	277.0 ± 124.0	[8]
400 µg (vaginal)	10		80.0 ± 27.0	165.0 ± 86.0	
400 µg (vaginal)	10	human serum	91.5 ± 82.0	445.9 ± 428.7	[35]
400 µg (vaginal)	10		51.0 ± 20.2	427.1 ± 235.5	
400 µg (buccal)	10		84.0 ± 81.9	264.8 ± 170.7	
400 µg (rectal)	10		19.5 ± 14.2	202.2 ± 195.7	
800 µg slow release (oral)	11	human serum	96.0 ± 168	78.8 ± 51.1	[36]
400 µg (sublingual)	9		30.0 ± 0.0	580 ± 178.1	
400 µg (vaginal)	10		102.0 ± 72.0	117.7 ± 42.1	
400 µg (vaginal)	14	human serum	120 ± 90	262.6 ± 201.1	[37]
400 µg (vaginal) misoprostol tablets moistened with 3 mL of saline solution	14		56.8 ± 45.6	1092.4 ± 1538.3	
400 µg (vaginal) misoprostol tablets moistened with 3 mL of 5% acetic acid	14		52.5 ± 37.16	703.4 ± 464.6	
800 µg (sublingual)	10	human plasma	30.0	1140 (817–2060)	[38]
800 µg (buccal)	8		30.0	229 (140 –1160)	
800 µg (oral)	10	human serum	20.7 ± 11.16	2683.0 ± 1216.1	[39]
800 µg (sublingual)	10		42.72 ± 24.9	2439.1 ± 1156.7	
800 µg (buccal)	10		78.48 ± 37.44	1361.1 ± 343.6	
600 µg (rectal)	10	human serum	40.5 ± 15.9	184.0 ± 64.5	[40]
600 µg (oral)	10		18.0 ± 8.8	327.9 ± 102.9	

## Data Availability

Not applicable.
